# Knowledge, Attitudes, and Practices Survey on Hypertensive Nephropathy Among Hypertensive Patients in Xinjiang, China: A Cross-Sectional Study

**DOI:** 10.1007/s11606-025-10079-7

**Published:** 2026-01-13

**Authors:** Wen Jiang, Xiaojuan Zhang, Mengru Wang, Wenbo Yang, Xiaoguang Yao, Nanfang Li

**Affiliations:** 1 Hypertension Center of People’s Hospital of Xinjiang Uygur Autonomous Region, Xinjiang Hypertension Institute, National Health Committee Key Laboratory of Hypertension Clinical Research, Key Laboratory of Xinjiang Uygur Autonomous Region “Hypertension Research Laboratory; Xinjiang Clinical Medical Research Center for Hypertension (Cardio-Cerebrovascular) Diseases, Urumqi, China; 2 Traditional Chinese Medicine Department of Xinjiang, Uygur Autonomous Region, Urumqi, China

**Keywords:** hypertension, hypertensive nephropathy, knowledge, attitude, health behavior, cross-sectional studies, China

## Abstract

**Background:**

This study aimed to assess the knowledge, attitudes, and practices (KAP) related to hypertensive nephropathy among patients diagnosed with hypertension in the Xinjiang of China.

**Methods:**

A cross-sectional survey was conducted between April 1 and April 30, 2024, in Xinjiang, China. Data were collected using a structured questionnaire designed to capture demographic characteristics and evaluate patients' KAP toward hypertensive nephropathy. Participants were individuals with a confirmed diagnosis of hypertension.

**Results:**

A total of 687 hypertensive patients were included in the final analysis, yielding a response rate of 98%. Among them, 58% were male. The majority of respondents (43%) had been diagnosed with hypertension for three years or less. Their median [q25, q75] knowledge, attitude, and practice scores were 6 (2,10) (possible range: 0–32), 24 (23,26) (possible range: 6–30), and 25 (18,31) (possible range: 9–45), respectively. The structural equation modeling (SEM) showed that knowledge had direct effects on attitude (β = 0.42, *P* < 0.001) and practice (β = 2.87, *P* < 0.001). Meanwhile, attitude had a direct effect on practice (β = -0.31, *P* < 0.001). Furthermore, knowledge indirectly affected practice through attitude (β = -0.13, *P* = 0.005).

**Conclusion:**

Patients diagnosed with hypertension in Xinjiang exhibited limited knowledge, generally positive attitudes, but suboptimal health practices regarding hypertensive nephropathy. To improve clinical outcomes, educational interventions targeting hypertension-related renal complications should be prioritized, with a focus on enhancing patient knowledge as a means to foster more effective self-care behaviors.

**Supplementary Information:**

The online version contains supplementary material available at 10.1007/s11606-025-10079-7.

## INTRODUCTION

Hypertensive nephropathy represents a significant subtype of chronic kidney disease (CKD), predominantly arising from persistent, inadequately controlled hypertension.^1^ Pathologically, this condition is characterized by arteriolosclerosis, progressive deterioration of renal function, and ultimately, in advanced stages, progression to end-stage renal disease (ESRD).^2^ The global burden of kidney disease affects approximately 850 million individuals, with hypertension established as the second most prevalent etiology after diabetes mellitus.^3^ This burden is expected to intensify as populations worldwide continue to age and hypertension prevalence increases. By 2019, the global age-standardized incidence of hypertension-related CKD had reached approximately 19.5 per 100,000 population, reflecting an 18% increase since 1990.^4^ In high-income countries, nearly one-fifth of individuals with hypertension develop CKD, while data from the United States demonstrate that approximately 36% of hypertensive adults manifest CKD, compared to merely 10% among normotensive individuals.^5,6^

The burden of hypertensive nephropathy is particularly substantial in China. In 2019, an estimated 5.58 million individuals suffered from hypertension-induced CKD, resulting in over 70,000 deaths and approximately 1.69 million disability-adjusted life years (DALYs) lost.^7^ Moreover, the disease exhibits notable demographic and geographic disparities, disproportionately affecting older adults, males, rural populations, and residents of western regions.^7,8^ Xinjiang province represents a particularly concerning case, with an age-standardized prevalence estimated at 307 per 100,000—significantly exceeding the national average observed in the more developed eastern provinces.^7^

In Xinjiang, the prevalence of hypertension is particularly high, especially among rural and minority populations. This is partly due to dietary habits such as high salt intake, frequent consumption of milk tea and preserved foods, as well as relatively low levels of physical activity. At the same time, uneven distribution of healthcare resources and limited access to specialized services have resulted in lower rates of blood pressure control compared to other regions. Since hypertension is a major risk factor for chronic kidney disease, people in Xinjiang may face a disproportionately high burden of renal complications. Cultural and linguistic barriers may also affect patients’ understanding of their condition and adherence to treatment. In addition, the findings from Xinjiang help fill an important gap in national research by representing the realities of underserved and marginalized populations, which are often overlooked in broader surveys.

The Knowledge-Attitude-Practice (KAP) theoretical framework has been widely implemented to investigate and influence health-related behaviors across various contexts.^9^ This structured approach typically employs questionnaires to systematically assess individuals' awareness, perceptions, and behavioral responses regarding specific health conditions.^10^ The framework operates on the fundamental assumption that knowledge positively influences attitudes, which subsequently guide practices.^11^ Researchers have extensively applied this model in studies focusing on prevention and management of chronic diseases, including hypertension and diabetes.^12–14^ In chronic disease management, KAP surveys are commonly used to identify patients’ misconceptions about diseases and medications, detect unfavorable attitudes that may hinder adherence, and evaluate actual health behaviors such as diet, medication use, and follow-up visits. Evidence has shown that improvements in patients’ knowledge and attitudes are associated with better medication adherence and blood pressure control, which in turn reduce the risk of cardiovascular events and kidney damage. Therefore, conducting a KAP survey among hypertensive patients provides not only baseline information but also an evidence base for designing targeted interventions and health education programs. However, research specifically examining hypertensive patients' understanding and management of hypertensive nephropathy remains limited—particularly in ethnically diverse, underrepresented regions such as Xinjiang.

This study aims to assess the knowledge, attitudes, and practices related to hypertensive nephropathy among individuals with hypertension in Xinjiang, China. Additionally, it seeks to identify sociodemographic and clinical determinants associated with variations in KAP scores. Through systematic evaluation of patients' understanding and self-management behaviors, this research will help identify critical gaps in knowledge and practice, thereby informing the development of targeted health education and intervention strategies. Such efforts may ultimately contribute to enhanced self-management capabilities, delayed disease progression, and more equitable prevention and control of hypertensive kidney disease in high-risk populations.

## METHODS

### Study Design and Participants

This cross-sectional survey was conducted between April 1 and April 30, 2024, in Xinjiang, China. Participants were recruited from the Hypertension Center at Xinjiang People’s Hospital. The study protocol was reviewed and approved by the Ethics Committee of Xinjiang People’s Hospital (Approval No. KY2022080904), and written informed consent was obtained from all individuals prior to participation.

Eligible participants met the following inclusion criteria: individuals aged 18 years or older; a clinical diagnosis of hypertensive nephropathy in accordance with the 2023 Chinese Guidelines for the Management of Hypertension in Patients with Chronic Kidney Disease, which includes a history of hypertension with a history of hypertension, evidence of renal impairment such as microalbuminuria or an estimated glomerular filtration rate (eGFR) below 60 mL/min/1.73 m^2^, and exclusion of other possible causes of kidney disease, evidence of microalbuminuria, or an estimated glomerular filtration rate (eGFR) below 60 mL/min/1.73 m^2^, Patients with coexisting conditions such as diabetes were not excluded, as the aim of this study was not to clinically attribute nephropathy to a single cause but rather to evaluate hypertensive patients’ knowledge, attitudes, and practices regarding hypertensive nephropathy; and receipt of standardized antihypertensive therapy during hospitalization, such as treatment with angiotensin-converting enzyme inhibitors (ACEIs), angiotensin II receptor blockers (ARBs), or sodium-glucose co-transporter-2 (SGLT-2) inhibitors.

Exclusion criteria comprised the presence of end-stage renal diseases of other etiologies, including diabetic nephropathy and polycystic kidney disease; severe cardiovascular or cerebrovascular conditions, such as acute myocardial infarction or stroke; psychiatric disorders or cognitive impairments that could compromise the accuracy of questionnaire responses; and unwillingness to provide informed consent.

### Sample Size Determination

The required sample size was estimated using the standard formula for calculating proportions in cross-sectional studies:

$$n = \left({Z}^{2} \times p \times \left(1-p\right)\right) / {d}^{2}$$

where Z represents the critical value at a 5% level of significance (Z = 1.96), p denotes the anticipated proportion of the target population, and d is the acceptable margin of error, set at 0.05. Given the absence of precise prior prevalence data, the expected proportion was conservatively set at 50% to maximize sample size and ensure statistical robustness. Based on these parameters, the minimum required sample size was calculated to be 384.^15^

## PROCEDURES

The questionnaire used in this study was developed based on a review of relevant published literature. Following its initial design, a pilot survey was conducted among 32 randomly selected hypertensive patients to assess the clarity, reliability, and applicability of the instrument. The preliminary results yielded a Cronbach’s alpha coefficient of 0.8894, indicating good internal consistency. Based on feedback and issues identified during the pilot phase, the questionnaire was revised and refined to enhance its structure, clarity, and respondent comprehension.

The final version of the questionnaire, administered in Chinese, comprised four sections. The first section collected demographic information through 12 items addressing age, sex, education, occupation, and other relevant characteristics. The second section assessed knowledge of hypertensive nephropathy using 16 items. Each item provided three response options: “Very familiar,” “Heard of it,” and “Unclear,” with scores assigned on a scale from 0 to 2 points, resulting in a total possible knowledge score ranging from 0 to 32. The third section evaluated attitudes through 6 items rated on a five-point Likert scale, with responses ranging from “Strongly disagree” to “Strongly agree,” corresponding to scores from 1 to 5; the cumulative attitude score ranged from 6 to 30. The fourth section addressed practice behaviors through 9 items, also rated on a five-point Likert scale based on the frequency or degree of engagement in specific actions, yielding a total practice score between 9 and 45.

A convenience sampling method was employed. The formal survey was carried out in April, targeting hypertensive inpatients admitted for hypertension management in the specialized Hypertension Department, which is a dedicated inpatient unit for the evaluation and management of patients with difficult-to-control or complicated hypertension. Patients with adequate smartphone literacy were invited to complete the questionnaire by scanning a WeChat-based QR code. A total of 702 questionnaires were collected, of which 687 were deemed valid and included in the final analysis.. The survey team consisted of five trained undergraduate students in their third year of study, all of whom received uniform instruction on the study protocol, questionnaire content, and standard operating procedures for assisting respondents.

Questionnaire administration was conducted through a digital survey platform. Upon completion, investigators immediately reviewed each submission for completeness and accuracy of key items. All valid questionnaires were assigned a unique identification number corresponding to each participant. To ensure data accuracy and minimize potential information bias, data entry was performed independently by two separate teams using a double-entry method. The research team then conducted cross-validation to resolve discrepancies and further examined the questionnaires for internal consistency, logical coherence, and completeness prior to formal statistical analysis.

### Statistical Analyses

Data analysis was performed using STATA version 14.0 (StataCorp LLC, College Station, TX, USA). Continuous variables were expressed as means and standard deviations (SD), while categorical variables were presented as frequencies and percentages [n (%)]. The Shapiro–Wilk test was used to assess the normality of continuous data. For comparisons across groups, one-way analysis of variance (ANOVA) was applied to normally distributed variables with homogeneous variance, whereas the Kruskal–Wallis test was employed for non-normally distributed data. Spearman’s rank correlation analysis was conducted to examine the relationships among knowledge, attitude, and practice (KAP) scores. Furthermore, structural equation modeling (SEM) was used to explore the direct and indirect pathways linking knowledge, attitude, and practice. Model fit was evaluated using standard indices, including the root mean square error of approximation (RMSEA), comparative fit index (CFI), Tucker–Lewis index (TLI), and incremental fit index (IFI). A two-sided P-value of less than 0.05 was considered statistically significant.

## RESULTS

### Questionnaire Quality

Initially, a total of 702 samples were collected. The following samples were excluded: 12 were excluded due to incomplete comorbidity information, and 15 were excluded because they inconsistently selected both “None” and one or more comorbidities. The final valid data consisted of 687 cases, with an effective rate of 97.86%. The Cronbach’s α coefficient for the overall questionnaire was 0.9015, for knowledge 0.9435, for attitude 0.9178, and for practice 0.8850. KMO = 0.940 (*P* < 0.001).

### Demographic Information on Participants

The study included 687 hypertensive patients (58% male; mean age 51.44 ± 13.40 years; BMI 26.30 [23.84, 28.60]) from Xinjiang, with notable demographic variations influencing KAP outcomes. Most participants were Han Chinese (71%), married (87%), urban residents (83%), and had ≤ 3 years of hypertension duration (43%). Their median [q25, q75] knowledge, attitude, and practice scores were 6(2,10), 24(23,26), and 25(18,31), respectively. Females exhibited significantly higher knowledge (median [q25, q75]: 6 [2.5, 11] vs. 5 [2, 9]; *P* = 0.007) and practice scores (29 [21.5, 33] vs. 22 [17, 29]; *P* < 0.001) compared to males. Education level strongly correlated with KAP performance: participants with master’s degrees achieved markedly higher knowledge (14 [12, 22]), attitude (29 [25.5, 30]), and practice scores (33.5 [29.5, 39.5]) than those with junior high school education or below (all *P* < 0.001). Medical staff outperformed other occupations across all domains (knowledge: 19.5 [12, 26]; attitude: 28.5 [24, 30]; practice: 34 [28, 39]; *P* < 0.001), while farmers scored lowest in knowledge (3 [1, 9]). Urban residents demonstrated higher knowledge (6 [2, 10] vs. 3 [1, 10]; *P* = 0.016) than suburban/rural counterparts. Longer hypertension duration (3–5 years) correlated with elevated knowledge (7 [4, 11]; *P* = 0.001) and practice scores (28 [21, 33]; *P* = 0.002), whereas ≥ 10 years of antihypertensive medication use paradoxically linked to lower practice scores (22 [17, 29]; *P* < 0.001) (Table 1).

**Table 1:** Baseline Characteristics

*N* = 687	N (%)	Knowledge score		Attitude score		Practice score	
		**Median [q25, q75]**	**P**	**Median [q25, q75]**	**P**	**Median [q25, q75]**	**P**
**Total score**		6(2,10)		24(23,26)		25(18,31)	
**Gender**			0.007		0.199		< 0.001
Male	399(58.08)	5(2,9)		24(23,28)		22(17,29)	
Female	288(41.92)	6(2.5,11)		24(23,25)		29(21.5,33)	
**Age(years old)[18 ~ 88]**	51.44 ± 13.40						
**Ethnicity**			0.877		0.525		0.324
Han	488(71.03)	6(2,10)		24(23,26)		25(18,31)	
Uyghur	114(16.59)	5(1,12)		24(23,26)		25(19,31)	
Kazakh	34(4.95)	6(2,12)		24(22,24)		23(17,29)	
Other	51(7.42)	4(1,10)		24(23,29)		22(18,29)	
**BMI[16.53 ~ 38.42]**	26.30(23.84,28.60)						
**Education**			< 0.001		0.001		< 0.001
Junior high school or below	160(23.29)	4(2,8.5)		24(23,26)		23.5(17.5,30)	
Senior high school/Technical secondary school	174(25.33)	4(2,9)		24(23,25)		23(17,30)	
Associate degree/Bachelor’s degree	333(48.47)	6(3,10)		24(23,26)		25(19,31)	
Master's degree or above	20(2.91)	14(12,22)		29(25.5,30)		33.5(29.5,39.5)	
**Marital status**			< 0.001		0.604		0.004
Single	58(8.44)	12(6,21)		24(22,28)		29.5(20,37)	
Married	599(87.19)	5(2,9)		24(23,26)		24(18,31)	
Divorced/Widowed	30(4.37)	5(2,10)		24(23,25)		26(19,32)	
**Occupation**			< 0.001		< 0.001		< 0.001
Medical staff	38(5.53)	19.5(12,26)		28.5(24,30)		34(28,39)	
Farmer	54(7.86)	3(1,9)		24(23,24)		25(18,29)	
Enterprise employee	137(19.94)	5(2,8)		24(23,25)		24(18,30)	
Public servant (e.g., government agencies/institutions)	140(20.38)	5(2,9)		24(23,29)		22(17,28.5)	
Worker (e.g., construction industry)	12(1.75)	6(2.5,12)		23(21,24.5)		25(20,31.5)	
Freelancer	49(7.13)	5(2,10)		24(23,25)		29(20,32)	
Self-employed/Businessperson	68(9.9)	5(2,8)		24(23,26)		23.5(18,30)	
Retired	165(24.02)	6(2,9)		24(23,25)		25(20,32)	
Other, please specify	24(3.49)	6(3,11.5)		24(24,26)		23(17.5,27.5)	
**Place of residence**			0.016		0.463		0.877
Urban	573(83.41)	6(2,10)		24(23,26)		25(18,31)	
Suburban/rural	114(16.59)	3(1,10)		24(23,25)		23.5(18,31)	
**Duration of hypertension**			0.001		0.001		0.002
≦3 years	294(42.79)	4(1,10)		24(23,25)		24(18,31)	
3–5 years	98(14.26)	7(4,11)		24(23,25)		28(21,33)	
5–10 years	118(17.18)	6(3,10)		24(23,26)		26.5(18,31)	
≧10 years	177(25.76)	6(3,10)		24(23,30)		23(17,29)	
**Duration of taking antihypertensive medication**			0.015		< 0.001		< 0.001
≦3 years	339(49.34)	5(2,10)		24(23,25)		24(18,32)	
3–5 years	97(14.12)	7(4,11)		24(23,26)		28(23,34)	
5–10 years	108(15.72)	5(2.5,10)		24(23,25)		26(18,30)	
≧10 years	143(20.82)	6(2,10)		24(24,30)		22(17,29)	
**Family history of hypertension**			0.647		0.618		0.200
Yes	143(20.82)	6(2,10)		24(23,26)		25(18,30)	
No	143(20.82)	5(2,11)		24(23,27)		25(18,33)	
**Comorbidities (multiple choices allowed)**							
None	480(69.87)						
Cerebrovascular disease	79(11.5)						
Heart disease	65(9.46)						
Diabetes	80(11.64)						
Kidney disease	29(4.22)						
Peripheral vascular disease	10(1.46)						
Other	7(1.02)						

### Knowledge, Attitude, and Practice

The distribution of knowledge dimension identified significant knowledge gaps among hypertensive patients in Xinjiang. While 52% were aware of hypertension diagnostic criteria (K1), critical deficits persisted in understanding renal complications. Only 8% recognized that prolonged hypertension induces renal vascular damage and fibrosis (K3), with 68% expressing uncertainty. Strikingly, 73% were unaware that HN’s pathological features overlap with other kidney diseases (K6), and 78% could not distinguish between benign and malignant nephrosclerosis subtypes (K8). Therapeutic knowledge was particularly poor: 57% doubted the renal protective role of ACEI/ARBs (K10), and 74% lacked awareness of aldosterone antagonists’ benefits (K11). Additionally, 55% were uncertain about HN’s contribution to end-stage renal disease (K5), and 49% questioned early-stage reversibility with treatment (K12) (Table 2). Responses to the attitude dimension showed that only 21% strongly agreed that long-term use of ACEI/ARB antihypertensive drugs can reduce the risk of hypertensive nephropathy (A3), only 23% strongly agreed that the longer the history of hypertension the more likely it is to develop proteinuria (A4), and only 25% strongly agreed that hypertensive patients are a high-risk group for hypertensive nephropathy (A1) (Table 3). Responses to the practice dimension showed that 37% never limit protein intake (P7), 32% never limit salt intake (P3), 28% never actively control weight (P1) (Table 4).

**Table 2:** Distribution of Knowledge Dimension Responses

Knowledge	N(%)		
	**Very familiar**	**Heard of it**	**Not sure**
**K1. Hypertension is defined as: without the use of antihypertensive drugs, office blood pressure measured on multiple occasions on different days shows systolic blood pressure (SBP) ≥ 140 mmHg and/or diastolic blood pressure (DBP) ≥ 90 mmHg**	265(38.57)	360(52.4)	62(9.02)
**K2. Hypertension is the second most common cause of chronic kidney disease (CKD). It accelerates CKD progression and increases the risk of cardiovascular disease and mortality**	65(9.46)	300(43.67)	322(46.87)
**K3. Long-term elevated blood pressure can cause pathological changes in intrarenal arterioles and small arteries, leading to arterial lumen narrowing, secondary ischemic damage to the renal parenchyma, and eventually glomerulosclerosis, tubular atrophy, and interstitial fibrosis—this is known as hypertensive nephropathy**	57(8.3)	164(23.87)	466(67.83)
**K4. Clinical manifestations of hypertensive nephropathy include:**			
(1) Increased nighttime urination	48(6.99)		
(2) Low specific gravity urine	256(37.26)		
(3) Mild to moderate proteinuria	383(55.75)		
(4) Progressive decline in glomerular filtration rate (GFR)	/		
**K5. Hypertensive nephropathy is an important cause of end-stage renal disease (ESRD)**	52(7.57)	258(37.55)	377(54.88)
**K6. The pathological features of hypertensive nephropathy are not unique to hypertensive renal injury and may be similar to or confused with those of metabolic syndrome, diabetes, IgA nephropathy, etc**	40(5.82)	147(21.4)	500(72.78)
**K7. Renal biopsy is an important method for the definitive diagnosis of hypertensive nephropathy and can help rule out certain other kidney diseases**	51(7.42)	301(43.81)	335(48.76)
**K8. There are two types of kidney damage caused by hypertension: benign arteriolar nephrosclerosis and malignant arteriolar nephrosclerosis**	37(5.39)	113(16.45)	537(78.17)
**K9. Hypertensive patients usually have a history of hypertension for more than 5 years before the appearance of proteinuria**	47(6.84)	207(30.13)	433(63.03)
**K10. ACEI/ARB class antihypertensive drugs (e.g., sartans) are the first-line recommendation in treatment guidelines for patients with hypertensive nephropathy, and they offer greater renal protection compared to other antihypertensive drugs**	49(7.13)	247(35.95)	391(56.91)
**K11. Aldosterone levels are closely related to the progression of hypertensive nephropathy. Aldosterone receptor antagonists can help improve the degree of kidney damage**	42(6.11)	137(19.94)	508(73.94)
**K12. The early stages of hypertensive nephropathy can be alleviated—and even reversed—with active treatment**	54(7.86)	295(42.94)	338(49.2)
**K13. The fundamental goal of treating hypertensive nephropathy is to protect target organ function, reduce cardiovascular and cerebrovascular complications, and lower mortality risk**	53(7.71)	315(45.85)	319(46.43)

**Table 3:** Distribution of Attitude Dimension Responses

Attitude	Strongly agree	Agree	Neutral	Disagree	Strongly disagree
A1. Hypertensive patients are a high-risk group for hypertensive nephropathy	171(24.89)	429(62.45)	67(9.75)	14(2.04)	6(0.87)
A2. Dietary treatment and lifestyle improvement are fundamental measures for hypertensive patients to prevent hypertensive nephropathy	196(28.53)	445(64.77)	34(4.95)	9(1.31)	3(0.44)
A3. Long-term use of ACEI/ARB antihypertensive drugs can reduce the risk of hypertensive nephropathy	144(20.96)	360(52.4)	167(24.31)	10(1.46)	6(0.87)
A4. The longer the history of hypertension, the more likely it is to develop proteinuria	156(22.71)	396(57.64)	117(17.03)	14(2.04)	4(0.58)
A5. The blood pressure control target for hypertensive nephropathy patients should be as individualized as possible	219(31.88)	398(57.93)	55(8.01)	11(1.6)	4(0.58)
A6. Standard blood pressure measurement and recording should be conducted to assess the risk of diseases related to blood pressure	205(29.84)	437(63.61)	33(4.8)	9(1.31)	3(0.44)

Items were scored on a five-point Likert scale in the positive direction (1 = strongly disagree/poorer knowledge or unfavorable practice, 5 = strongly agree/better knowledge or favorable practice). Higher scores indicate more positive responses

**Table 4:** Distribution of Practice Dimension Responses

Practice	Always	Often	Sometimes	Rarely	Never
P1. Actively control weight	67(9.75)	180(26.2)	141(20.52)	107(15.57)	192(27.95)
P2. Engage in moderate-intensity physical exercise, with at least 150 min of total exercise time per week	42(6.11)	103(14.99)	168(24.45)	188(27.37)	186(27.07)
P3. Limit salt intake	47(6.84)	143(20.82)	178(25.91)	97(14.12)	222(32.31)
P4. Increase intake of fruits and vegetables, while limiting high-potassium foods	42(6.11)	174(25.33)	217(31.59)	143(20.82)	111(16.16)
P5. Quit smoking and drinking	353(51.38)	63(9.17)	58(8.44)	50(7.28)	163(23.73)
P6. Maintain a regular lifestyle, get enough sleep, and keep an optimistic and cheerful mood	63(9.17)	159(23.14)	272(39.59)	158(23)	35(5.09)
P7. Limit protein intake	29(4.22)	98(14.26)	206(29.99)	99(14.41)	255(37.12)
P8. Eat less food high in cholesterol (e.g., animal brains and offal) and saturated fats	50(7.28)	130(18.92)	215(31.3)	133(19.36)	159(23.14)
P9. Select appropriate antihypertensive drugs based on urine protein levels, kidney function, organ damage, and complications	48(6.99)	144(20.96)	186(27.07)	126(18.34)	183(26.64)

Items were scored on a five-point Likert scale in the positive direction (1 = never/poorer practice, 5 = always/better practice). Higher scores indicate more positive responses

### Correlation Analysis

Correlation analysis indicated significant positive correlations between knowledge and attitude (r = 0.1468, *P* < 0.001), as well as practice (r = 0.4612, *P* < 0.001). However, the correlation between attitude and practice was not statistically significant (Table 5).

**Table 5:** Correlation Analysis

	**Knowledge**	**Attitude**	**Practice**
Knowledge	1		
Attitude	0.1468 (*P* < 0.001)	1	
Practice	0.4612 (*P* < 0.001)	−0.1001 (*P* = 0.008)	1

### SEM Analysis

**Figure 1:**
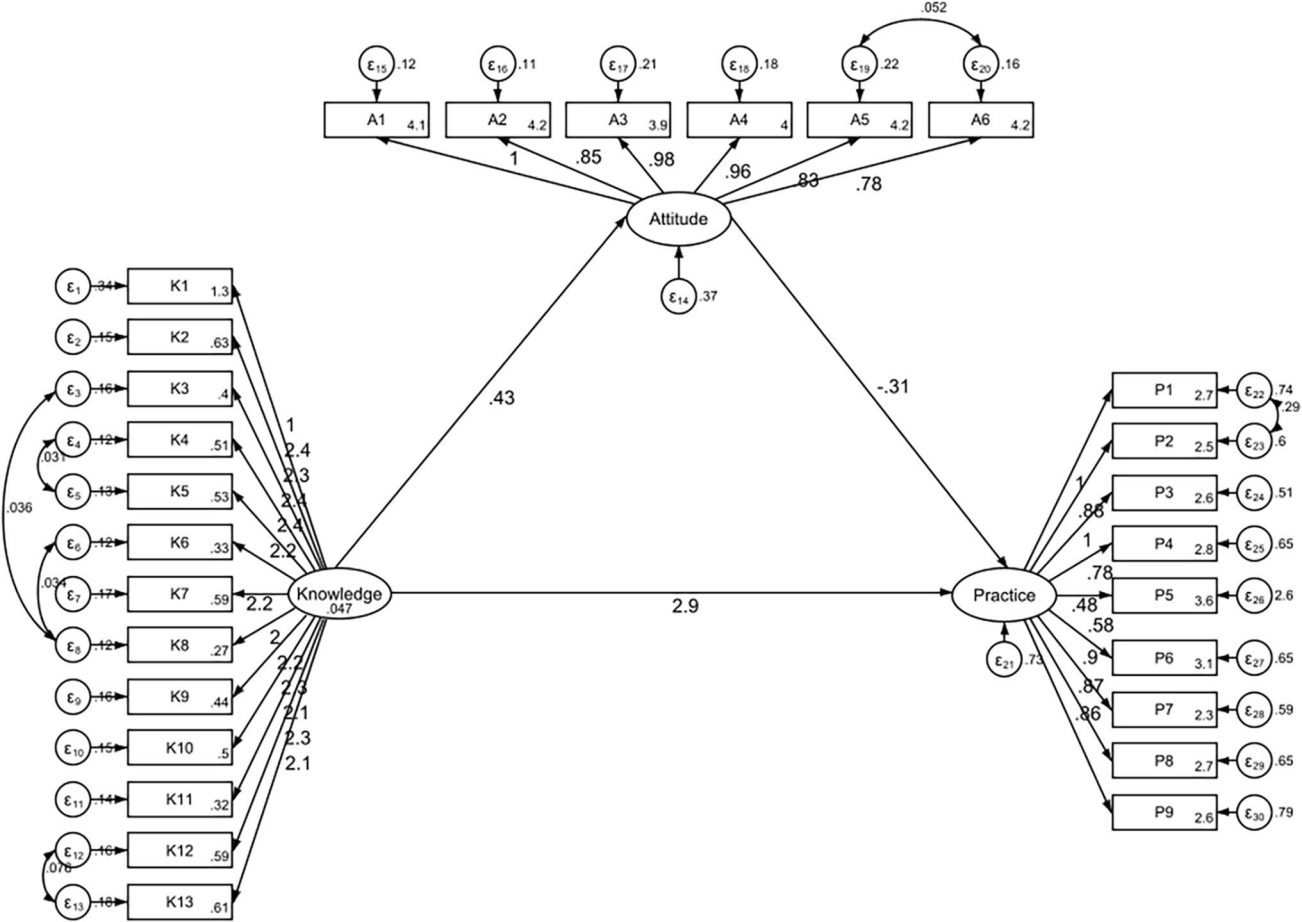
SEM model.

The SEM demonstrate a highly favorable model fit indices (RMSEA value: 0.070, SRMR value: 0.060, TLI value: 0.906, and CFI value: 0.915), suggesting a well-fitting model (Table S1), and the effect estimates between the various paths have been presented (Table S2). Analysis of the direct and indirect effects of the model showed that knowledge had direct effects on attitude (β = 0.42, *P* < 0.001) and practice (β = 2.87, *P* < 0.001). Meanwhile, attitude had a direct effect on practice (β = −0.31, *P* < 0.001). Furthermore, knowledge indirectly affected practice through attitude (β = −0.13, *P* = 0.005) (Table S3 and Fig. 1).

## DISCUSSION

Patients with hypertension in Xinjiang demonstrated limited knowledge, generally positive attitudes, yet suboptimal practices regarding the prevention and management of hypertensive nephropathy. These findings highlight the need for targeted educational interventions that not only enhance disease-specific knowledge but also bridge the gap between attitudes and self-management behaviors in clinical practice. These findings carry particular weight in Xinjiang, where medical resources are unevenly distributed and health education efforts are often insufficient. In addition, the region’s multiethnic composition means that cultural and language differences may limit the effectiveness of conventional patient education and communication. Conducting a KAP survey in this context helps to identify the specific knowledge gaps and behavioral challenges faced by patients, which can then inform more practical and culturally appropriate intervention strategies. Beyond local relevance, the results also provide evidence that may be useful for improving chronic disease management in other underserved and multiethnic regions.

Previous research has indicated that the effectiveness of hypertension management and the prevention of related renal complications largely depend on patients’ health literacy and their sustained engagement in self-care behaviors.^16,17^ In this study, hypertensive patients in Xinjiang generally demonstrated limited understanding of hypertensive nephropathy, moderate levels of preventive attitudes, and insufficient behavioral practices. The relationships among these domains suggest that while some degree of awareness exists, it is often not accompanied by actionable health behavior, highlighting important disconnects between intention and practice. These patterns, although somewhat predictable—such as higher knowledge among women, city dwellers, and medical professionals—are still important to demonstrate. They provide region-specific evidence that helps identify priority populations for targeted education and intervention.

The first theme that emerged from our analysis centered on the foundational role of knowledge. The overall knowledge performance was relatively poor, especially in understanding disease pathophysiology, pharmacological strategies, and diagnostic criteria. Participants with higher education levels and medical backgrounds tended to exhibit more accurate knowledge, which is consistent with previous studies showing that formal education and occupational exposure positively influence disease comprehension.^18^ However, among the broader population, there was a clear gap in recognizing both the existence and the progression of hypertensive nephropathy, including uncertainty about early warning signs and treatment benefits. Such gaps are not unique to this setting but have also been observed in studies conducted in rural areas of Southeast Asia, where culturally tailored education programs were found to improve comprehension and care adherence.^18,19^ These findings suggest an urgent need to design interventions that not only convey information but do so in a contextually appropriate and comprehensible manner. For example, incorporating visual materials and community-based explanations delivered in local languages may help reach populations with limited biomedical familiarity.

The second theme addressed attitudes toward hypertensive nephropathy. Although most participants endorsed prevention-oriented beliefs, these attitudes did not translate into consistent practices. Structural equation modeling further highlighted a somewhat paradoxical pattern: while knowledge had a positive influence on attitude, the latter showed an unexpected negative association with behavior. This discrepancy challenges traditional assumptions within the KAP framework and suggests that attitudes, though superficially positive, may lack the depth or confidence necessary to motivate change. Interestingly, our SEM analysis confirmed that knowledge was positively associated with both attitude (β = 0.42, *p* < 0.001) and practice (β = 2.87, *p* < 0.001). However, contrary to the traditional KAP framework, attitude showed a negative association with practice (β = –0.31, *p* < 0.001), leading to a small negative indirect effect of knowledge on practice through attitude (β = –0.13, *p* = 0.005). This suggests that, in this population, positive attitudes may not consistently translate into healthier behaviors, possibly due to cultural or structural barriers. Similar incongruities between belief and behavior have been noted in chronic disease management studies conducted in other low-resource settings, where participants often express support for preventive action but do not engage in it due to competing life demands, perceived futility, or lack of structural support.^20,21^ It is plausible that participants in this study viewed the idea of kidney protection as important in theory but struggled with its practical implications in the absence of supportive mechanisms.

The third theme pertained to behavioral practices, which were the weakest domain of performance. Participants showed limited engagement in recommended lifestyle modifications such as dietary regulation, physical activity, or personalized medication adjustments. These findings align with earlier studies in comparable contexts, where chronic disease self-management remains underdeveloped despite growing awareness efforts.^22,23^ The data also revealed variation in practice patterns based on gender, occupation, and education, suggesting that structural determinants shape individuals’ capacity to implement what they know. Previous work from similar multiethnic regions has emphasized the role of environmental and socioeconomic constraints in shaping chronic disease behaviors, pointing to barriers such as time poverty, food insecurity, and lack of trust in health providers.^24,25^ In Xinjiang, additional layers such as linguistic diversity, uneven health infrastructure, and cultural preferences may further limit the feasibility of daily health behavior change. Moreover, although item P6 on maintaining an optimistic and cheerful mood is not a direct medical intervention, evidence suggests that positive psychological attitudes may enhance self-management behaviors and improve quality of life among patients with chronic diseases.^26^

The interconnectedness of the KAP dimensions became increasingly evident through the combined lens of our correlation and modeling analyses. Knowledge appeared to serve as a prerequisite for action, while attitudes, though present, lacked the consistency to act as reliable mediators. Practices, ultimately, were shaped by a confluence of individual understanding and external support, pointing to the importance of multilevel alignment between patient capacity and system design. These findings underscore the limitations of relying solely on individual-level interventions and suggest that knowledge-based strategies must be situated within a broader ecology of behavioral enablers. Health system responsiveness, provider engagement, and resource accessibility are just as critical as patient awareness in producing meaningful outcomes.^27,28^

To enhance the current situation, efforts should begin with the development of targeted health education campaigns that address specific knowledge gaps identified in this study. These should be coupled with provider training programs that encourage consistent, culturally sensitive communication and emphasize personalized care plans. In addition, system-level policy adjustments are needed to institutionalize behavioral support into routine hypertension management, including flexible service delivery models, patient reminders, and multidisciplinary care teams. Health authorities might also consider piloting integrated chronic care initiatives that consolidate medication management, lifestyle coaching, and family involvement under a unified framework. Only by addressing the interplay of knowledge, attitude, and practice in a coordinated manner can sustainable improvements in patient outcomes be realized.^29–31^

This study has several limitations. First, its cross-sectional design limits the ability to infer causal relationships between knowledge, attitudes, and practices. Second, the use of self-reported questionnaires may introduce response bias, as participants might overestimate or underestimate their actual behaviors. In addition, some items in the practice section, such as P7 on protein restriction, reflect traditional patient education but may not fully align with current KDIGO recommendations, which emphasize individualized dietary guidance. This limitation should be considered when interpreting the findings. Third, since the study was conducted in a single region and limited to patients who attended healthcare facilities, the findings may not be generalizable to all hypertensive populations in other areas or settings.

In conclusion, patients with hypertension in Xinjiang demonstrated insufficient knowledge, relatively positive attitudes, and suboptimal self-management practices regarding hypertensive nephropathy, with knowledge exerting both direct and indirect influences on behavior through attitude. Given these findings, clinical strategies should prioritize educational interventions that strengthen patients’ understanding of hypertensive nephropathy to ultimately improve adherence to preventive practices.

## Supplementary Information

Below is the link to the electronic supplementary material.

Supplementary file1 (DOCX 16 KB)

## Data Availability

All data generated or analysed during this study are included in this published article.
