# Supplementary material for: Knowledge, Attitudes, and Practices Survey on Hypertensive Nephropathy Among Hypertensive Patients in Xinjiang, China: A Cross-Sectional Study
Source: J Gen Intern Med. 2026 Jan 13;41(7):1899–908. doi: 10.1007/s11606-025-10079-7 (PMC13176424; doi:10.1007/s11606-025-10079-7)
Supplement: Supplementary file 1 — Supplementary file1 (DOCX 16 KB) [file 11606_2025_10079_MOESM1_ESM.docx]

**Supplementary table 1. SEM fit indicators**

| Indicators | Reference | Results |
| --- | --- | --- |
| RMSEA | <0.08 Good | 0.070 |
| SRMR | <0.08 Good | 0.060 |
| TLI | >0.8 Good | 0.906 |
| CFI | >0.8 Good | 0.915 |

**Supplementary table 2. SEM total effect estimates**

|  |  | **Estimate** | **P>\|z\|** |
| --- | --- | --- | --- |
| **Structural** |  |  |  |
| Attitude |  |  |  |
|  | Knowledge | 0.43 | 0.001 |
| Practice |  |  |  |
|  | Attitude | -0.31 | <0.001 |
|  | Knowledge | 2.87 | <0.001 |
| **Measurement** | |  |  |
| K1 | Knowledge | 1 |  |
| K2 | Knowledge | 2.40 | <0.001 |
| K3 | Knowledge | 2.30 | <0.001 |
| K4 | Knowledge | 2.39 | <0.001 |
| K5 | Knowledge | 2.41 | <0.001 |
| K6 | Knowledge | 2.15 | <0.001 |
| K7 | Knowledge | 2.19 | <0.001 |
| K8 | Knowledge | 1.97 | <0.001 |
| K9 | Knowledge | 2.20 | <0.001 |
| K10 | Knowledge | 2.29 | <0.001 |
| K11 | Knowledge | 2.08 | <0.001 |
| K12 | Knowledge | 2.25 | <0.001 |
| K13 | Knowledge | 2.13 | <0.001 |
| A1 | Attitude | 1 |  |
| A2 | Attitude | 0.85 | <0.001 |
| A3 | Attitude | 0.98 | <0.001 |
| A4 | Attitude | 0.96 | <0.001 |
| A5 | Attitude | 0.83 | <0.001 |
| A6 | Attitude | 0.78 | <0.001 |
| P1 | Practice | 1 |  |
| P2 | Practice | 0.88 | <0.001 |
| P3 | Practice | 1.04 | <0.001 |
| P4 | Practice | 0.78 | <0.001 |
| P5 | Practice | 0.48 | <0.001 |
| P6 | Practice | 0.58 | <0.001 |
| P7 | Practice | 0.90 | <0.001 |
| P8 | Practice | 0.87 | <0.001 |
| P9 | Practice | 0.86 | <0.001 |

**Supplementary table 3. Analysis of direct and indirect effects**

| Model paths |  | Total effects | | Direct Effect | | Indirect effect | |
| --- | --- | --- | --- | --- | --- | --- | --- |
|  |  | β (95% CI) | P | β (95% CI) | P | β (95% CI) | P |
| Asum <- |  |  |  |  |  |  |  |
|  | Ksum | 0.42(0.18,0.67) | 0.001 | 0.42(0.18,0.67) | 0.001 |  |  |
| Psum <- |  |  |  |  |  |  |  |
|  | Asum | -0.31(-0.43,-0.19) | <0.001 | -0.31(-0.43,-0.19) | <0.001 |  |  |
|  | Ksum | 2.74(2.05,3.42) | <0.001 | 2.87(2.16,3.58) | <0.001 | -0.13(-0.22,-0.04) | 0.005 |
